# Immigrants, health, and the impact of COVID-19: A narrative review

**DOI:** 10.12688/f1000research.130085.2

**Published:** 2023-11-20

**Authors:** Khadijah Angawi

**Affiliations:** 1 Department of Health Services and Hospital Administration; Faculty of Economics and Administration,, King Abdulaziz University, Jeddah, 80200, Saudi Arabia

**Keywords:** Immigrants, COVID-19, Health, impact, access to health care, socioeconomic, socio-psychological, effective measures.

## Abstract

While the COVID-19 pandemic has gravely challenged health systems globally, countries that host a large number of refugees are finding themselves even more burdened as providing preventive and curative services to refugees, and, migrants has proved to be a challenging task. The aim of this narrative review is to discuss the impact COVID-19 pandemic on immigrants, and seek to understand how COVID-19 affects provision of health services, access to health care and the socioeconomic situation. Like any other health challenge, COVID-19 has also left migrants susceptible to adverse outcomes, both directly and indirectly. Several factors limit their ability to avoid infections, access healthcare, and cope with socio-psychological impacts. In addition, undocumented immigrants or people living on short-term visit visas do not have full access to healthcare services in most countries. It is evident that COVID-19 has also influenced these workers leaving them jobless or receiving low wages or no pay, hence, this has hugely impacted the remittance and economic situation in their country. Extending access to healthcare to the entire immigrant population, irrespective of their legal status, is the cornerstone of an effective response to counter the COVID-19 pandemic.

## Introduction

While the COVID-19 pandemic has gravely challenged health systems globally, countries that host a large number of refugees are finding themselves even more burdened as providing preventive and curative services to refugees has proved to be a challenging task. ^1^ Quarantine and lockdown measures taken by the government, while adequate, can lead to undesirable results for immigrants. These undesirable results are primarily due to behavioral patterns by immigrants in response to the public health interventions and policies implemented by the government. Refugees tend to flee disease control centers and thus become carriers of diseases with a high potential of infecting other healthy people. ^2^ Research shows that migrating epicenters impose a net of public health consequences by putting others at risk of infections in host countries. ^2^^,^^3^

Migration and health maintain a multidimensional and dynamic relationship. Health and wellbeing are key drivers for migration as migrants move from poor to rich countries for better health condition, but such a relocation also impacts migrants’ health. ^4^ The three main population groups targeted for a public health intervention to mitigate the spread of emerging outbreaks and their negative effects: the people living in the source area; the fleeing population leaving the source area; and the people traveling from infected regions to other areas. ^4^

Behavioral differences between destination and origin or spread of health conditions have also been found to be associated with circular migration, which affects the health status of others. ^5^ Apart from being a public health concern, epidemics can also lead to an economic crisis. ^4^ A large number of internal migrant workers are laid off from work due to government policies enacted to curtail the spread of disease. Thus, such people are often left trapped in cities. Since a majority of such workers are underpaid and barely earn over subsistence wages, they do not possess any other means of protecting their economic interests other than their jobs. ^4^

COVID-19, although a tremendous challenge, is not the first pandemic to highlight the fundamental disparities in public health management. As recently as 2009, there had been an opportunity in the form of the H1N1 Influenza pandemic to study and understand the inequalities faced by vulnerable population groups, similar to those observed during COVID-19. Both indicate eventually poor health and socioeconomic outcomes for disadvantaged people groups such as migrants. ^6^ Moreover, limited access to preventive medical care, bigger households, inability to comply with work from home instructions even when ill, and reliance on the use of public transportation have put immigrants at higher risk of H1N1. ^6^^–^^8^ Apart from inequalities in just clinical outcomes, the H1N1 pandemic personified the disparity in pandemic preparedness, response, and recovery for normal and disadvantaged population groups, including the immigrant communities. Despite such obvious findings from the H1N1 pandemic, the disaster preparedness for immigrant communities has largely remained inadequate and ineffective. ^6^

Although, the effects of migration on the spread of the disease have been the subject of many research studies, epidemics’ effects on immigrants are an important aspect that is still largely understudied. The aim of this narrative review is to discuss the impact of the COVID-19 pandemic on immigrants, and seek to understand how COVID-19 affects provision of health services, access to health care and the socioeconomic situation. Capturing these different impacts is vital to develop effective measures to overcome these challenges among immigrants. The focus of this research was a number of countries including Saudi Arabia (SA), the United Arab Emirates (UAE), Turkey, Norway, China, Colombia, Australia, and the United States (US), which is known for having a large number of foreign immigrants. Since the COVID-19 outbreak, providing preventive and curative care for immigrants has proven to be difficult, adding to the burden on these nations that already host large numbers of immigrants.

### Scope of the review

This article provides a thorough analysis of the COVID-19 pandemic’s diverse consequences on a range of population categories, including migrants, refugees, legal immigrants, and illegal immigrants. Even though these groups experience similar obstacles, they also have different stressors and require different support networks.

Refugees: The study examines how the pandemic has affected countries with considerable refugee populations, creating difficulties in providing preventive and therapeutic healthcare services.

Immigrants: Despite being an essential component of global community, immigrants are vulnerable due to COVID-19. The discussion explores the ways in which this health crisis exposes immigrants to a range of unfavourable consequences, such as infection risk, access to healthcare, and sociopsychological effects.

Migrants: The paper focuses on the complex relationships that exist between health, infectious disease transmission, and migration. The current study focuses in particular on how diseases such as COVID-19 affect migrant workers, both domestically and abroad, analysing their socioeconomic conditions.

Undocumented Immigrants: The study reveals the challenges that undocumented immigrants encounter when attempting to obtain healthcare services. It also examines how the pandemic has affected their employment, income, and financial security.

Through examining the effects of COVID-19 on these disparate but related population groups, this paper seeks to offer a comprehensive understanding of their difficulties. It also emphasises how important it is to provide healthcare to everyone, regardless of immigration status.

## Materials and methods

Different databases such as Google Scholar and PubMed were searched for this narrative review. All articles published on the topic of immigrants and COVID-19 were searched by using search terms such as ‘Immigrants,’ ‘Migrants,’ ‘COVID-19’, ‘Sars-CoV2’ ‘immigration,’ and ‘Refugees’. While conducting a detailed and updated search, complete references and citations (where available), relevant to the topic, were downloaded into Endnote X9 (referencing program), for further assessment. Published studies in the English language and conducted in developing or developed countries were included. All the studies published during 2020-2021 were reviewed. A snowball sampling technique was utilized for the identification of eligible studies. As the first step, researcher reviewed the literature, retrieved studies abstract and checked the titles which matched the inclusion criteria. Later, references of the included studies were also scanned for the identification of relevant studies. Furthermore, full-text articles highlighted the impact of current pandemic of covid-19 on immigrants were reviewed.

### The impact of the COVID-19 pandemic on immigrants

Like any other health challenge, COVID-19 has left migrants susceptible to adverse outcomes, both directly and indirectly. Their capacity to fend off illnesses and deal with socio-psychological effects is restricted by a number of factors. Poor living and working conditions, a lack of access to health care, xenophobia, a lack of knowledge of the local environment, a lack of community networks, and a lack of consideration for cultural and linguistic diversity by service providers are just a few of the major shortcomings in host countries. ^9^ It becomes virtually impossible to practice social distancing and good hygiene in camp and non-camp settings for people groups such as refugees or internally displaced people. ^10^ Apart from this, most workers engaged in informal economy also often experience an abrupt loss of income.

With limited access to healthcare, the complexities of poverty, and a threat of legal ramifications, immigrant communities in the US find themselves at an elevated risk of contracting SARS-CoV-2 and developing COVID-19. About 37.2% or more than 500,000 immigrants in the US are undocumented. In the state of Texas alone, around 32% of undocumented immigrants live below the poverty line. In comparison, 64% of them do not carry any medical insurance, thus have very few options to cater to their medical needs. ^11^ Similarly, Turkey is home to the largest number of refugees in the world. There were approximately four million people living as refugees in Turkey in 2020. ^12^ A vast majority of refugees in Turkey (around 98%) live in urban centers, big, crowded cities like Istanbul, Gaziantep, and Hatay. Such a huge number of refugees in already overpopulated cities puts immense pressure on these cities already strained public health infrastructure. It limits their ability of effective health responses, including COVID-19. ^12^ Additionally, during the first wave of COVID-19, unemployment among foreign workers in Gulf countries became widely prevalent, resulting in a reduction in their income levels as well as in their ability to remit money back home. ^13^ A study reported that more than 52% of foreign workers experienced a significant loss of income during the initial wave of COVID-19, owing to the closure of different industries. ^13^

While not many home evictions were reported during the first wave, as a consequence of governments’ protective policies, rising unemployment did result in a strain on expatriate workers’ ability to pay rent. Those facing wage cuts or outright termination were affected the most. This economic disruption has also affected their socio-psychological condition. In the absence of a formal mechanism at an institutional level, such disruption may also lead to physical and mental health issues such as depression, stress, isolation, and suicidal tendencies. ^14^ While about 90% of migrant workers did have health insurance during the first wave (excluding those who were unemployed or on visit visas), the rising domestic rate of unemployment in the Gulf cities has made expatriate workers express their fear of being unable to afford health insurance in the near future. Such an outcome will eventually influence their decision to stay and increase their vulnerability during the rest of the pandemic. While countries like SA and the UAE did reportedly offer free healthcare services at their public health institutions to undocumented migrants who presented COVID-19 symptoms, the effectiveness of such state-led interventions needs to be examined further, especially concerning their accessibility. ^13^

Opportunities for looking for another job or the ability to move internally or internationally for people working in exploitative conditions even before the pandemic are quite limited. Their situation is further aggravated by the small amount of savings they have. In countries where migration status is linked to one’s employer and job, the shutdown of a workplace may result in irregularities. ^15^ Moreover, migrants who pay for the renewal of their residence and work permits themselves may not be able to afford them anymore if they lose some part of their income. Sectors such as agriculture, construction, logistics, healthcare, municipal services, etc., have largely remained active during the pandemic and contain a disproportionate number of migrant/expatriate workers. People employed in such sectors are unable to work from home. Consequently, close physical proximity to co-workers and customers, inability to access private transport and an overall lack of protective equipment make such professions riskier. ^15^

Various studies have reported various socioeconomic and health dipartites faced by undocumented immigrants as a result of COVID-19. There is a high prevalence of COVID-19, anxiety, depression, and avoidance of seeking health care reported among immigrants. In terms of socioeconomic impacts of COVID-19 lockdown, undocumented immigrants faced reductions in working hours which impacted their income. Not to mention, the surplus of food and housing security that has accumulated due to lost income ever since the COVID-19 lockdown began. ^16^^,^^17^ Another study found a high prevalence of poor health conditions including depression, reduced quality of life, and increased levels of alcohol misuse were associated with the fear of COVID-19. ^18^ A cross-sectional study found a high prevalence of COVID-19 in low-skilled migrants working in supermarkets, which might be the cause of the spread of disease in the community. Additionally, poor housing conditions and less physical distancing were attributable to high prevalence of COVID-19 in these workers. ^19^^,^^20^ A study conducted in Norway found that migrants living there experience a higher burden of COVID-19 infections and hospitalization compared to non-immigrants. However, immigrants in the study showed a high level of adherence to most of the health preventive measures for COVID-19. ^19^

### Provision of healthcare

Accessibility and affordability of healthcare during the COVID-19 pandemic are extremely crucial. ^21^^,^^22^ An early diagnosis and monitoring of patients with COVID-19 play a critical role in the eventual outcome for the patient and further transmission of the disease in the rest of the community. ^22^ Most vulnerable immigrants are already either under-insured or un-insured altogether and therefore depend on free clinics, or public health centers. ^2^ Such centers have a very limited ability to provide testing, management, and follow-up services as they are often underfunded. Furthermore, lack of access to preventive medicines increases the risk of developing underlying conditions such as hypertension, obesity, and diabetes. These comorbidities are known to inflict more severe manifestations of COVID-19. ^21^^,^^23^^,^^24^ In addition to this, an immigrant’s mode of entry into the countries like US might put them at risk for excessive stress due to fears of poverty, trauma, and poor social support. This leads to mental health problems such as PTSD, anxiety, and depression. These stress factors may potentially worsen during the pandemic, especially for those who are at higher of SARS CoV-2 or at a higher risk of losing their job and thus have limited healthcare resources. ^11^ Many immigrants are already at an increased risk of exposure to SARS CoV-2 since their economic condition dictates that they continue their work, which often requires physical presence at the workplace and involves face-to-face interactions. ^6^ Immigrants often live in large, multigenerational households or with multiple roommates. Consequently, one infected person in such a setup exposes every other household member, including the elderly, and immunosuppressed. ^25^ With the COVID-19 pandemic intensifying across the cities in the Gulf, such as Dubai and Jeddah, the role of public health institutions and medical services has become even more pivotal. Countries like SA and the UAE have committed to providing free healthcare services to COVID-19 patients, regardless of their immigration status. Although these countries have allowed migrant workers access to their public hospitals, migrants, particularly undocumented immigrants and their families, are still quite likely to face health insecurity due to unemployment and full medical insurance coverage. ^13^

### Access to healthcare

Undocumented immigrants or people living on short-term visit visas do not have full access to healthcare in most countries. This means they are often not entitled to free medical treatment and thus have to bear the cost out of their own pockets. ^26^ Besides, evidence indeed suggests that people from poor and marginalized communities have limited access to healthcare and that their socio-economic conditions tend to affect the impact of COVID-19 on their livelihoods. The rate of COVID-19 infections, hospitalization or deaths are far higher than what their ratio among the general population might suggest. Similar is the case with people facing food and financial insecurity. ^27^ Fear of being reported to immigration authorities and deportation also reduces or influences undocumented immigrants’ eagerness to seek assistance in the onset of COVID-19 symptoms and have subsequent screening, testing, contact tracing. Likewise, another qualitative study reported that immigrants are faced with legal, and financial obstacles affecting their access to healthcare. ^28^ Additionally, immigrants were found to encounter unjust discrimination by the hospitals’ points of entry (e.g., guards, clerks, public health staff), which effectively prevents immigrants from receiving services because of their prejudice or ignorance. ^28^^,^^29^

The intersection of class, race, and status form the bedrock where specific patterns of vulnerability for migrants can be found. Migrants, while overrepresented in lower-income groups, are often excluded from welfare programs and face other forms of discrimination, all the while fearing arrest and deportation. Despite the lack of adequately disaggregated data, there is some evidence present to suggest that so far immigrants have suffered disproportionately throughout the pandemic in certain locations. ^30^ A qualitative study conducted on Venezuelan migrants in Columbian cities has highlighted that migrants faced legal and financial obstacles that impacted their ability to access healthcare. ^28^ The main barrier for accessing healthcare in these host countries was lack of having legal migratory status. Venezuelan migrants seeking asylum were under a temporary visa permit and not eligible, and this prevented them from getting access to medical care. ^28^ The COVID-19 situation has resulted in a higher prevalence of economic informalities and health disparities among immigrants.

Adherence to their specific cultural customs, unfamiliarity with locally recommended preventive measures, and an overwhelming dependence on informal communication channels are some of the reasons behind seemingly odd behaviors that migrants adopt, which later put them and their community at an elevated risk of disease transmission. Coupled with their living and traveling conditions, these factors make immigrant communities more vulnerable to the direct health impacts of COVID-19. ^31^ It has also been noted that digital illiteracy creates communication and language barriers for gaining access to healthcare. ^32^^,^^33^ Since local authorities often do not have accurate data on the number and distribution of migrants within their jurisdiction, such communities are left partially or, in some cases, even fully excluded from public health programs. Consequently, it becomes very difficult to collect accurate information about affected individuals in such communities or to monitor them to trace out the source of an outbreak. Instead, authorities have to rely on close surveillance of entire populations for effective tracing. ^15^

### Socioeconomic challenges faced by immigrants

With more than 23% of the total immigrant population in the US comprising of undocumented migrants, an insight into the socio-economic perspective of the prevailing COVID-19 pandemic bears utmost importance. ^34^ While initially only international travellers and their close contacts were affected by SARS-CoV-2/COVID-19 in the US, later on, it withered many disadvantaged communities and continues to do so. As has been the case in any other historical pandemic, socio-economic factors have strongly influenced vulnerability to and eventual health outcomes of COVID-19 as well. Thus, it is not difficult to predict that low-income and vulnerable communities in the US will almost certainly be disproportionately affected. It is already evident from the emergence of certain “hot-spots”; areas have shown unexpected and higher rates of COVID-19 related deaths amongst impoverished minority populations, and this might be due to the higher prevalence of comorbidities which in turn are a consequence of lack of timely and adequate access to healthcare owing to unequal socio-economic factors. ^11^

Likewise, immigrant workers are more vulnerable to the socioeconomic hardships of COVID-19. A study by Karim *et al.* reported that immigrants from Bangladesh faced various financial and socio-economic crises due to COVID-19 with respect to loss of jobs due to lockdowns. Surprisingly, the economy of Bangladesh is highly dependent on remittance that is continuously flowing into the country by their immigrant workers living abroad. However, COVID-19 has influenced these workers leaving them jobless or receiving low wages or no pay, hence, this has hugely impacted the remittance and economic situation in their country. Many restrictions were imposed by the Bangladesh government not allowing the immigrants to leave the host country and they suffered from mental pressure and social discrimination. Many Bangladeshis immigrant workers died from COVID-19, among them workers were mostly doctors. ^35^ Similarly, this pandemic highlights the ethnic and socio-economic inequalities among different native-migrants living in the UK. ^36^ These inequalities have a negative impact on migrants’ economic well-being. Fears of high unemployment rates and excessive financial strains among immigrant communities as a consequence of the COVD-19 pandemic are not unfounded. Only 57% of people among the entire immigrant population currently carry private medical insurance. Loss of job directly equates with loss of access to healthcare for themselves and their families, as they will no longer be able to pay insurance premiums. ^11^ Fears of food insecurity are also growing in impoverished communities due to the disruptions caused by the COVID-19 pandemic in the global supply chain.

Despite the challenges and consequences of immigration, it is important to acknowledge the valuable assets and benefits immigrants bring. Immigrants are recognized as valuable assets across nations, contributing significantly to their adopted homelands. In the United States, 69% of immigrants hold essential “critical infrastructure jobs”. These immigrants fulfil crucial roles in the country’s infrastructure, healthcare, manufacturing, food, and safety sectors. Many immigrants had significant contributions on the frontline in combating COVID-19. Their dedication is vital for the smooth functioning of various societal sectors. Prioritizing their health and welfare is crucial, serving not only humanitarian reasons but also benefiting nations worldwide from a strategic standpoint. ^37^

### Measures that need to be taken

While the entire global community has been gravely affected by SARS-CoV-2, it has placed marginalized people in particular at an elevated risk of infection and experiencing severe manifestations of COVID-19. Hence, it has become imperative to act at all levels of government, be it local, state/provincial, or national, to ensure adequate and timely intervention to improve not only their access to healthcare but also their legal and economic status as well. All COVID-19 related relief programs in the future must aim to expand their inclusivity and widen the safety-net health systems in all disadvantaged communities. Provision of free and widely available SARS-CoV-2 screening, and testing is key. All such future programs must also make changes at the policy level for curtailing the backbreaking costs of healthcare for those who are unable to afford private health insurance. However, in the long run, it will be essential to improve the primary healthcare infrastructure for better diagnosis, treatment, and control of comorbidities in high-risk populations. This will help greatly enhance the eventual outcomes in vulnerable migrant communities in the event of a future pandemic. Alongside these health measures, it is also important to relieve the economic burden of immigrants as well by creating safety nets in their employment contracts against sudden lay-offs. Moreover, rapid dissemination of culturally and linguistically apt public health messages among at-risk communities will help them improve their general awareness, preparedness and response. Healthcare must be extended to, in fact, prioritized for all disadvantaged communities, including immigrants and irrespective of their legal status, to mitigate the devastating, inequitable health and financial costs repeatedly and predictably borne by such communities during every epidemic disease outbreak.

While countries like the UAE and SA have seemingly extended free healthcare in their public hospitals/care centers for migrant/expatriate workers who presented symptoms of COVID-19, particularly to those who were undocumented, further in-depth analysis of ease-of-access and efficacy of such government-led programs is required to paint a broader picture. Extending access to healthcare to the entire immigrant population, irrespective of their legal status, is the cornerstone of an effective response to counter the COVID-19 pandemic. While some countries were already providing universal health coverage to all of their population, including migrants, many have tried to remove the obstacles that block immigrants’ access to COVID-19 screening and testing. Many governments are using the formal and informal communication channels popular amongst immigrant communities and try to engage organizations and individuals well known and trusted among such communities. Migrants cannot be successfully included in any screening, contact tracing, and healthcare provision regimen until overcoming trust barriers. One way of doing this can be erecting a barricade between healthcare and immigration enforcement. This can help in reducing their fears of arrest and deportation in the event of discovery of their immigration status while they seek medical assistance. Immigrants are known to trust non-governmental healthcare providers whom they know more than government institutions. Therefore, adequate resources must be allocated to ensure continuity of such services, which will, in turn, encourage immigrants to seek timely help.

## Data Availability

No data are associated with this article.

## References

[1] Ginsburg C Bocquier P Béguy D : Association between internal migration and epidemic dynamics: an analysis of cause-specific mortality in Kenya and South Africa using health and demographic surveillance data. *BMC Public Health.* 2018;18(1):1–14. 10.1186/s12889-018-5851-5 PMC6062880 30049267

[2] Eiset AH Wejse C : Review of infectious diseases in refugees and asylum seekers-current status and going forward. *Public Health Rev.* 2017;38:22. 10.1186/s40985-017-0065-4 29450094 PMC5810046

[3] Mesnard A Seabright P : Escaping epidemics through migration? Quarantine measures under incomplete information about infection risk. *J. Public Econ.* 2009;93(7-8):931–938. 10.1016/j.jpubeco.2009.05.001

[4] Khanna A : Impact of migration of labour force due to global COVID-19 pandemic with reference to India. *J. Health Manag.* 2020;22(2):181–191. 10.1177/0972063420935542

[5] Siegel M : *Migration and health. Routledge Handbook of Migration and Development.* Routledge;2020;221–231.

[6] Blendon RJ Koonin LM Benson JM : Public response to community mitigation measures for pandemic influenza. *Emerg. Infect. Dis.* 2008;14(5):778–786. 10.3201/eid1405.071437 18439361 PMC2600239

[7] Quinn SC Kumar S Freimuth VS : Racial disparities in exposure, susceptibility, and access to health care in the US H1N1 influenza pandemic. *Am. J. Public Health.* 2011;101(2):285–293. 10.2105/AJPH.2009.188029 21164098 PMC3020202

[8] Yu H Feng Z Uyeki TM : Risk factors for severe illness with 2009 pandemic influenza A (H1N1) virus infection in China. *Clinical infectious diseases: an official publication of the Infectious Diseases Society of America.* 2011;52(4):457–465. 10.1093/cid/ciq144 21220768 PMC3060897

[9] Liem A Wang C Wariyanti Y : The neglected health of international migrant workers in the COVID-19 epidemic. *Lancet Psychiatry.* 2020;7(4):e20-e. 10.1016/S2215-0366(20)30076-6 32085842 PMC7129812

[10] Sanderson D : *Coronavirus: Having fled crisis before, displaced people living in Africa’s cities are especially at risk.* Sydney: Kaldor Centre for International Refugee Law;2020;7.

[11] Clark E Fredricks K Woc-Colburn L : Disproportionate impact of the COVID-19 pandemic on immigrant communities in the United States. *PLoS Negl. Trop. Dis.* 2020;14(7):e0008484. 10.1371/journal.pntd.0008484 32658925 PMC7357736

[12] Özvarış ŞB Kayı İ Mardin D : COVID-19 barriers and response strategies for refugees and undocumented migrants in Turkey. *J. Migr. Health.* 2020;1-2:100012. 10.1016/j.jmh.2020.100012 34405167 PMC8352003

[13] Al-Ghalib Alsharif FL Malit FT Jr : Migration and The COVID-19 Pandemic in the Gulf: Konrad-Adenauer-Stiftung e. V. 2020. Reference Source

[14] Hayward SE Deal A Cheng C : Clinical outcomes and risk factors for COVID-19 among migrant populations in high-income countries: a systematic review. *J. Migr. Health.* 2021;3:100041. 10.1016/j.jmh.2021.100041 33903857 PMC8061095

[15] Guadagno L : Migrants and the COVID-19 pandemic: An initial analysis. 2020.

[16] Burton-Jeangros C Duvoisin A Lachat S : The Impact of the Covid-19 Pandemic and the Lockdown on the Health and Living Conditions of Undocumented Migrants and Migrants Undergoing Legal Status Regularization. *Front. Public Health.* 2020;8:596887. 10.3389/fpubh.2020.596887 33392134 PMC7772178

[17] Alali WQ Bastaki H Longenecker JC : Seroprevalence of SARS-CoV-2 in migrant workers in Kuwait. *J. Travel Med.* 2021;28(2):taaa223. 10.1093/jtm/taaa223 33283232 PMC7799017

[18] Hall BJ Zhao P Xiong MZ : Exploring correlates of depression, quality of life and alcohol misuse: a nationwide cross-sectional study of international migrants during the COVID-19 epidemic in China. 2021;11(3):e048012. 10.1136/bmjopen-2020-048012 PMC7959221 33722876

[19] Madar AA Benavente P Czapka E : COVID-19: access to information, level of trust and adherence to health advice among migrants in Norway. 2021. 10.1186/s13690-021-00764-4 PMC8725426 34983639

[20] Indseth T Grøsland M Arnesen T : COVID-19 among immigrants in Norway, notified infections, related hospitalizations and associated mortality: A register-based study. 2020;1403494820984026. 10.1177/1403494820984026 PMC7859570 33406993

[21] Hardy LJ Getrich CM Quezada JC : A call for further research on the impact of state-level immigration policies on public health. *Am. J. Public Health.* 2012;102(7):1250–1253. 10.2105/AJPH.2011.300541 22594736 PMC3477996

[22] Martinez O Wu E Sandfort T : Evaluating the impact of immigration policies on health status among undocumented immigrants: a systematic review. *J. Immigr. Minor. Health.* 2015;17(3):947–970. 10.1007/s10903-013-9968-4 24375382 PMC4074451

[23] Alegría M Cao Z McGuire TG : Health insurance coverage for vulnerable populations: contrasting Asian Americans and Latinos in the United States. *Inquiry: a journal of medical care organization, provision and financing.* 2006;43(3):231–254. 10.5034/inquiryjrnl_43.3.231 17176967 PMC2712944

[24] Commodore-Mensah Y Selvin E Aboagye J : Hypertension, overweight/obesity, and diabetes among immigrants in the United States: an analysis of the 2010-2016 National Health Interview Survey. *BMC Public Health.* 2018;18(1):773. 10.1186/s12889-018-5683-3 29925352 PMC6011357

[25] Easthope H Liu E Burnley I : Changing perceptions of family: A study of multigenerational households in Australia. *J. Sociol.* 2017;53(1):182–200. 10.1177/1440783316635850

[26] Vearey JO Hui C Wickramage K : *Migration and health: current issues, governance and knowledge gaps. World Migration Report Geneva.* International Organization for Migration;2020;212–249.

[27] Devakumar D Shannon G Bhopal SS : Racism and discrimination in COVID-19 responses. *Lancet.* 2020;395(10231):1194. 10.1016/S0140-6736(20)30792-3 32246915 PMC7146645

[28] Zambrano-Barragán P Ramírez Hernández S Freier LF : The impact of COVID-19 on Venezuelan migrants’ access to health: A qualitative study in Colombian and Peruvian cities. *J. Migr. Health.* 2021;3:100029. 10.1016/j.jmh.2020.100029 34405183 PMC8352083

[29] D’Ignoti S : How coronavirus hits migrants and asylum seekers in Italy. *the new humanitarian.* 2020;16.

[30] Erdal MB Aden H Tellander E : Migrants and COVID-19 in Norway: Five reflections on skewed impacts. Beyond the COVID Curve, Migration PRIO blogs. 2020;6.

[31] Holguin F Moughrabieh MA Ojeda V : Respiratory health in migrant populations: a crisis overlooked. *Ann. Am. Thorac. Soc.* 2017;14(2):153–159. 10.1513/AnnalsATS.201608-592PS 28146384 PMC5427732

[32] Knights F Carter J Deal A : Impact of COVID-19 on Migrants’ Access to Primary Care: A National Qualitative Study. medRxiv. 2021:2021.01.12.21249692. 10.3399/BJGP.2021.0028 PMC8216266 33875420

[33] Bhandari D Kotera Y Ozaki A : COVID-19: challenges faced by Nepalese migrants living in Japan. *BMC Public Health.* 2021;21(1):752. 10.1186/s12889-021-10796-8 33874937 PMC8054259

[34] Batalova J Hanna M Levesque C : Frequently Requested Statistics on Immigrants and Immigration in the United States 2021. 2021 [cited 2021 Aug 18].

[35] Karim MR Islam MT Talukder B : COVID-19′s impacts on migrant workers from Bangladesh: In search of policy intervention. *World Dev.* 2020;136:105123. 10.1016/j.worlddev.2020.105123 32834394 PMC7395589

[36] Hu Y : Intersecting ethnic and native-migrant inequalities in the economic impact of the COVID-19 pandemic in the UK. *Res. Soc. Stratif. Mobil.* 2020;68:100528. 10.1016/j.rssm.2020.100528 32834346 PMC7351056

[37] Kerwin D Nicholson M Alulema D : *US foreign-born essential workers by status and state, and the global pandemic: Center for Migration Studies.* New York, NY;2020.

