# Supplementary material for: Immigrants, health, and the impact of COVID-19: A narrative review
Source: F1000Res. 2023 Nov 20;12:176. Originally published 2023 Feb 14. [Version 2] doi: 10.12688/f1000research.130085.2 (PMC10665605; doi:10.12688/f1000research.130085.2)
Supplement: Peer review history

# Reviewer report: Reviewer response for version 1

**DOI:** 10.5256/f1000research.142815.r215022

**Published:** 2023-11-22

**Authors:** Emanuele Politi

**Affiliations:** 1 KU Leuven, Leuven, Belgium

The provided review offers valuable insights into the impact of the COVID-19 pandemic on migrant populations, shedding light on the urgent need for targeted interventions and policies. To strengthen the article and maximize its impact, it is recommended that the author address the following points:

**1. Differentiate General and Migrant-Specific Risk Factors:**

- Clearly delineate which risk factors are applicable to the general population and which are unique to migrant communities (for a similar point, see Politi *et al.*, 2021 ^1^). This distinction is crucial for developing interventions that are both effective and appropriately tailored.
- Specify areas where structural, generalized interventions are necessary versus situations that require targeted, timely measures. This will contribute to a more nuanced understanding of the specific challenges faced by migrants during the pandemic.

**2. Identify Risk Factors Based on Migrant Categories:**

- Further elaborate on the different categories of migrants, such as undocumented immigrants,  transit migrants, resettled migrants, and seasonal workers. Investigate whether certain risk factors are more pronounced in specific migrant subgroups.
- Recognize the diversity within migrant populations and tailor recommendations accordingly. By understanding the unique challenges faced by each subgroup, policymakers can develop more effective and inclusive strategies.

**3. Contextualize Analyses:**

- Clearly distinguish between contexts when analyzing the impact of the pandemic on migrant populations. Acknowledge that the experiences of the same category of refugees, for example, may vary significantly between different countries or regions.
- Discuss explicitly which reception, integration, and protection policies have proven most effective in reducing risk factors within specific institutional contexts. Highlighting successful models can guide the development of evidence-based policies in diverse settings.

**4. Extend Reflection Beyond the Acute Phase of the Pandemic:**

- Encourage the author to look beyond the immediate crisis and propose recommendations that can be applied to the current context while also anticipating future global crises. This forward-looking approach will enhance the resilience of support and protection networks for vulnerable migrant populations.
- Emphasize the importance of long-term, sustainable interventions that address systemic issues contributing to the vulnerability of migrant communities. By doing so, the article can offer valuable insights for policymakers and practitioners preparing for future challenges.

Incorporating these suggestions will not only enhance the overall structure and argumentation of the review but also contribute to its broader relevance and applicability. Wishing the author success in further developing this important contribution to the discourse on the impact of the COVID-19 pandemic on migrant populations.

Is the review written in accessible language?

Yes

Are all factual statements correct and adequately supported by citations?

Yes

Are the conclusions drawn appropriate in the context of the current research literature?

Partly

Is the topic of the review discussed comprehensively in the context of the current literature?

Yes

Reviewer Expertise:

Social psychology, migration and refugee studies, socio-ecological models, acculturation and integration

I confirm that I have read this submission and believe that I have an appropriate level of expertise to confirm that it is of an acceptable scientific standard, however I have significant reservations, as outlined above.

## References

[1] : The Impact of COVID-19 on the Majority Population, Ethno-Racial Minorities, and Immigrants. *European Psychologist* .2021;26(4) : 10.1027/1016-9040/a000460 298-309 10.1027/1016-9040/a000460

# Reviewer report: Reviewer response for version 1

**DOI:** 10.5256/f1000research.142815.r215027

**Published:** 2023-10-27

**Authors:** Jaime Ballard

**Affiliations:** 1 University of Minnesota, Minnesota, USA

Dr. Angawi presents a summary of research on the impact of the COVID-19 pandemic on immigrants’ socioeconomic wellbeing. The review focuses on countries with a large number of foreign immigrants, and concludes with measures that need to be taken in these countries to improve wellbeing.  

 While this topic is essential, the review currently does not include a sufficient description of the methodology or sufficient citation support for the factual statements.

- **Clarify the scope. **Both abstract and paper currently open by describing refugees, then later shifts to describing immigrants, migrants and undocumented immigrants. Each of these groups have different supports and may face different stressors. A more unified description of scope, particularly in the introduction, would help guide the reader.
- **Describe the methods.** Currently, the only description of methods is the mention that research in this narrative review focused on countries known for having a large number of foreign immigrants. A greater description of search engines, search terms, date ranges, and any other inclusion criteria is necessary for readers to understand what evidence is being summarized here.
- **Review and strengthen the evidence for factual statements.** Many factual statements do not have citations listed (i.e., “Most vulnerable immigrants are already either under-insured or un-insured altogether and therefore depend on free clinics, or public health centers.” And “While not many home evictions were reported during the first wave, as a consequence of governments’ protective policies, rising unemployment did result in a strain on expatriate workers’ ability to pay rent. Those facing wage cuts or outright termination were affected the most.”) In some cases, the citation listed does not provide sufficient evidence for the statement. Notably, the statement “Research shows that migrating epicenters impose a net of public health consequences by putting others at risk of infections in host countries” references a mathematical modeling article about what migration might be expected under different information conditions; it does not have any data that implies increased risk of infection. Similarly, “Refugees tend to flee disease control centers and thus become carriers of diseases with a high potential of infecting other healthy people” has no citation. In the United States, legal refugees must undergo a lengthy immigration process including extensive health screenings, and this statement is inaccurate. Globally, reviews of evidence find that there is little risk of spread of infectious diseases from refugees and asylum seekers to the host population.
  - Eiset and Wejse (2017 ^1^).
- **Consider reframing to not focus solely on the burdens or threats posed by immigrants.** I am concerned that the article describes the threat of disease from immigrant populations (and this section needs stronger evidence) and the burdens of providing preventive and curative services, and does not also describe the assets/benefits brought by immigrants. In the States, 69% of all immigrants work in essential critical infrastructure jobs. Supporting immigrant health and well-being is necessary for national wellbeing.
  - https://cmsny.org/wp-content/uploads/2020/05/US-Essential-Workers-Printable.pdf

Is the review written in accessible language?

Yes

Are all factual statements correct and adequately supported by citations?

No

Are the conclusions drawn appropriate in the context of the current research literature?

No

Is the topic of the review discussed comprehensively in the context of the current literature?

Partly

Reviewer Expertise:

Immigrant and refugee family well-being

I confirm that I have read this submission and believe that I have an appropriate level of expertise to confirm that it is of an acceptable scientific standard, however I have significant reservations, as outlined above.

## References

[1] : Review of infectious diseases in refugees and asylum seekers-current status and going forward. *Public Health Rev* .2017;38: 10.1186/s40985-017-0065-4 22 10.1186/s40985-017-0065-4 29450094PMC5810046

# Reviewer report: Reviewer response for version 1

**DOI:** 10.5256/f1000research.142815.r169377

**Published:** 2023-05-03

**Authors:** Jasmin Lilian Diab

**Affiliations:** 1 Department of Social Sciences, School of Arts and Sciences, Lebanese American University, Beirut, Lebanon

This article does a very good job in unpacking the COVID-19 experience through an intersectional migration lens -- particularly, it outlines challenges at this intersection adequately. The most important contribution of this piece, is its integration of a labor/economic angle.

 In the case studies and examples it unpacks, it does an excellent comparative job -- and largely succeeds at establishing parallels and trends across the intersection of migration, labour and health. 

 While the paper would have benefited from a more in depth look into one particular case study -- as well as a little bit of data collection in the form of qualitative interviews, it makes up for this in the rich references it collects. However, once again, data triangulation would have strengthened the piece.

Is the review written in accessible language?

Yes

Are all factual statements correct and adequately supported by citations?

Yes

Are the conclusions drawn appropriate in the context of the current research literature?

Yes

Is the topic of the review discussed comprehensively in the context of the current literature?

Yes

Reviewer Expertise:

Migration and Refugee Studies; Gender Studies; Conflict Studies

I confirm that I have read this submission and believe that I have an appropriate level of expertise to confirm that it is of an acceptable scientific standard.
